# The Vegetative Receptor-Vascular Reflex (VRVR) – A New Key to Regeneration

**DOI:** 10.3389/fphys.2020.547526

**Published:** 2020-09-23

**Authors:** Michael Ofner, Harald Walach

**Affiliations:** ^1^Institute of Pathophysiology and Immunology, Medical University of Graz, Graz, Austria; ^2^Department of Pediatric Gastroenterology, Poznan University of Medical Sciences, Poznań, Poland; ^3^Department of Psychology, Witten/Herdecke University, Witten, Germany; ^4^Change Health Science Institute, Berlin, Germany

**Keywords:** fascia, extracellular matrix, regeneration, vegetative receptor-vascular reflex, manipulative therapies

## Abstract

**Objective:**

We describe a potentially new physiological reflex path that has so far been neglected but which could be used for a novel therapeutic approach: The vegetative receptor-vascular reflex. This is a physiological response that starts from the connective tissue and influences the whole organism. We cross-fertilized various research areas with each other.

**Key Findings:**

The matrix or the connective tissue forms a passive reservoir of substrate for the growth and development of cells, and functions as the primordial communication system of all living systems. It contains a continuous network of cells, such as fibroblasts, along with protein bundles made up of collagen that support electrical exchange through piezoelectric effects. This archaic vegetative system surrounds all cells, including neurons, and can thus be viewed as the primordial coordinating system in every organism. It is very likely the basis for a reflex which we describe here for the first time: the vegetative receptor vascular reflex. We also indicate some potential practical applications and test procedures.

**Conclusion:**

The vegetative receptor vascular reflex describes the pathway from stimuli that originate in the connective tissue or the extracellular matrix toward organ systems. They might be chemical in nature or electrical via piezo-electric effects stimulating nerve endings, and thus can influence higher order processes such as regeneration or healing of tissue. Thus, this reflex lends itself to a novel therapeutic approach via certain types of manipulation of the connective tissue.

## Background and Objective

Cells are functional as single entities and thus are considered the basic element of all life processes. From that insight the idea has developed, at the latest since 1850 following the work of Virchow cells are also the sole pathological driver for disease processes (Uexküll and Wesiack, 1988; Meyer-Abich, 2010). Practically all therapeutic efforts aim at influencing cells behaving pathologically – by removing them through surgery, by influencing them pharmacologically, or by supporting them otherwise. Once cells function normally, so the theory goes, health is restored. This standard view neglects some important facts.

First, cells never function as single entities; rather, they are always in an organismic context, i.e., controlled through higher order and top-down control processes (Sonnenschein and Soto, 1999; Spencer et al., 2010; Hyland, 2011; Kim et al., 2011; Scholkmann et al., 2013; Capra and Luisi, 2014). Such control processes and feedback mechanisms have become ever more important in understanding the behavior of single cells and as potential therapeutic targets (Discher et al., 2017). Second, cells are embedded in the interstitium of the matrix, also called the extracellular matrix (ECM) that forms the connective tissue (Heine and Anastasiades, 1992). When it is surrounding muscles, this connective tissue is referred to as fascia. It is likely that bone tissue also belongs to this functional entity (Langevin and Huijing, 2009; Schleip et al., 2012; Bordoni et al., 2018; Bordoni, 2019). We often forget that the connective tissue not only surrounds and encapsulate organs, thus differentiating it from other tissue and fulfilling a support and mechanical function; it also surrounds all cells and thus forms one holistic system that allows for an exchange of information, substrate and transport of waste products and nourishment, fulfilling a communicative and exchange function (Langevin, 2006; Tozzi, 2015a,b). Due to the emphasis on the single cell as the subject and object of health and disease, the holistic properties of organic subsystems and the regulatory mechanisms that govern them have come under closer scrutiny only recently. In what follows, we wish to reverse the emphasis, pointing out the active role that connective tissue can play in sustaining and regaining health. We will describe facts already known about connective tissue and fascia, combine some points that are usually discussed in isolation, and add some speculations. As a consequence, we will introduce the vegetative receptor-vascular reflex (VRVR), the pathway from stimuli originating in the matrix or the connective tissue that can influence and coordinate behavior of organ systems and regeneration processes in the body. This reflex is sometimes seen in manipulative therapy of injuries and might have much wider applicability (Litscher et al., 2013a,b; Ofner et al., 2014, 2018).

We cross-fertilized findings reported in the literature with the focus of synthesizing material that is relevant for our question.

## Key Findings

### Cells, Their Environment, and Its Regulation

Single cellular organisms are in constant exchange with their environment. Ameba, paramecia and other single cell organisms approach anything that can serve as substrate for incorporation, expel what is toxic, avoid dangerous conditions by moving away, and move to locations where they find beneficial conditions (Fels, 2009). Thereby they adapt to their environment. Through the course of evolution single cells probably combined to form organisms because this helped them to both passively adapt to their environment, and to actively control their environment and produce the conditions ultimately conducive to their sustenance (Varela, 1979; Hands, 2015). Evolution can be seen as internalizing all those adaptations that the single cell organism used to adapt externally to its environment (Carlucci et al., 2011). For instance, the liquid milieu which a paramecium or an ameba moves in is now *within* the organism and is the interstitium of our cells. Instead of adapting *to* its physical or chemical properties, our organism meanwhile actively changes the chemical and physical properties of its internal milieu, for instance by keeping temperature constant within useful boundaries, keeping chemical and physical processes in balance, etc. Thereby, sensing and regulating has been ever more centralized, while the basic mechanisms are still in place. Thus, gradually, the earlier and more primitive sensing and regulating mechanisms of simple unicellular or multicellular organisms have been complemented by more sophisticated regulating and coordinating mechanisms.

### The Most Basic Regulatory Process: The Archaic Vegetative System (AVS)

Every single cellular organism has to regulate its internal state: it takes up substrate and expels waste and noxious products via the cell membrane and membrane proteins. Through the process of cells combining to multicellular organisms and eventually to animals that can sense and move, higher order regulation processes have been added. But the most basic regulatory system is still in place. If organisms become more complex, cells at different places of the organism might have different needs and metabolic situations. In case of flight those cells in charge of movement, for instance, because they are flagellated, will need more nutritional substrate and oxygen than those in charge of intake, which will then have to be comparatively silent. Even very simple organisms have to manage these feedback and regulation processes.

It is reasonable to assume that in simple organisms without any neural system these processes function via internal exchange processes, for instance chemical or electric gradients (Werb and Chin, 1998; Tseng et al., 2010). Chemical gradients that activate osmotic pressure operate via ion currents and thus, basically, through electric phenomena (Tseng and Levin, 2013). Thus, the primordial interstitial matrix of primitive multicellular organisms contains the most basic exemplar of a communication process in nuce within their interstitial milieu: chemical gradients and electric currents/voltage. We can assume that these organization processes are still in place even in higher organisms that have gradually developed more sophisticated coordination mechanisms, such as a nervous system. Let us therefore call this most archaic communication system the *archaic vegetative system*. Its function is to support the health and the operation of the cells by supplying cells with substrate for growth, signals for differentiation, and energy, and to allow for the disposal of waste products including apoptotic cells and cell turnover (Pischinger, 2004).

In a further evolutionary step, when cells combined to multicellular organisms, this archaic vegetative system differentiated into cells specialized for sensing, cells specialized for motion or contractility, cells specialized for nutrient uptake and metabolism and so forth. The first primitive nervous system developed, which is now known as the enteric nervous system or the oldest branch of the autonomic nervous system (Kulkarni et al., 2018). This is known, for instance in one of the simplest organisms, the hydra, which is about 650 million years old (Furness and Stebbing, 2018). It already contains a network of about 100 neurons that regulate both the movement of the tentacles which moves nutrition-containing water into the organism and the sensing of the nutrients to start the intake of nutrients through cell walls. This is an example of the first neuronal sensing and motor system which forms the basis of the very first rudimentary brain. This is now called the enteric nervous system, or “gut brain,” and is part of the autonomic nervous system (Mayer, 2011).

Note that in higher organisms that are endowed with centralized nervous systems and many kinds of receptors there will be a more sophisticated interaction with these additional systems, but the basic nature of this system is still in place: the provision of nutrients and oxygen to cells and the transport and excretion of waste products and toxic stuff via chemical gradients, osmotic pressure and ionic or electric currents.

### The Vegetative System or Autonomic Nervous System (ANS)

Once multicellular organisms have developed, they bundle their communicative needs up in dedicated nervous systems. This is in part vegetative in the sense that it supports all processes necessary for the individual to sustain life. One element of this vegetative nervous system is the enteric nervous system that controls, monitors and executes the provision of nutrients to the organism. In more differentiated organisms it controls the digestive tract (Berthoud and Neuhuber, 2000; Mayer, 2011; Furness and Stebbing, 2018; Powley et al., 2019). A second and better-known part of this vegetative nervous system is what we call the autonomic nervous system, with its two branches, the sympathetic and the parasympathetic system. This controls and coordinates all other vegetative functions such as breathing, cardiovascular activity, internal homeostasis and immunological integrity (Venables, 1991; Baylis et al., 1993; Hori et al., 1995; Maier et al., 1998; Berthoud and Neuhuber, 2000; Goehler et al., 2000; Barman, 2019; Mondelli and Vernon, 2019; Muzik and Diwadkar, 2019; Pace-Schott et al., 2019). These vegetative nervous systems can be seen as increasing specializations of communicative functions into dedicated systems that in very simple organisms are only supported by the interstitial matrix. This specialization is achieved by channeling the electric currents more effectively.

During the evolutionary process these cell agglomerations develop into specialized nerve cells and later on even into myelinated, i.e., faster conducting nerve cells. The benefit of this development is obvious: important regulative functions can be centralized, first in ganglia, later in brains, where the afferent information can be compared, weighed and submitted to decision algorithms, and effective regulatory action can be executed consciously and subconsciously (Spencer et al., 2004). This can be selective and produce various seemingly disparate actions that are nevertheless coordinated to meet certain goals. We can see this in our own vegetative system, where afferents from various parts of our body, relaying information about such different parameters as blood pressure, pH of the blood, oxygenation of the blood, availability of ATP, body temperature, immune status, nutritional status, to name but a few, are relayed to our brainstem and further on to the hypothalamus, where all this information is being integrated and submitted to regulatory algorithms such that efferent nerve fibers can adapt the respective status in their target organ, for instance by regulating the tonus of the vessels in certain body parts. In higher organisms, the central nervous system (see below) plays an even higher regulative role via its interaction with the autonomic nervous system (Al-Khazraji and Shoemaker, 2018).

Thus, in more complex organisms we have not only the archaic vegetative system that operates via the interstitial matrix, using chemical gradients, ionic and electric currents as afferents and efferents, but also specialized nerve fibers differentiated into different parts of a vegetative nervous system *in addition*. The more complex the organism has become, the more complex its vegetative system.

Note, one important logical and biological consequence of this situation: Nerve fibers, being specialized cell assemblies, also have their own connective tissue and their own archaic vegetative system and interact with it. The more specialized the vegetative system has become the more diversified the interactional options between this system and the archaic vegetative system. In a simple organism without a nervous system the electro-chemical gradient is the only communication system. In a complex organism compartmentalized and specialized situations in certain locations can be operative. This then produces specific actions via the vegetative system, and at the same time holistic and global information transfer might occur via the archaic vegetative system *plus* a variety of interactions of these two.

### The Central Nervous System (CNS)

In higher animals the nervous system has evolved and differentiated itself, in addition to the vegetative nervous system, into a central nervous system. This latter system mainly has the function of relaying sensory information from available senses afferently to the brain and efferently to execute actions that require muscles for motion. In higher animals and humans we call these voluntary movements. Because our consciousness seems to be mainly supported by the central nervous system (CNS), the CNS has become, historically speaking, the main target of scientific interest, while the autonomic or vegetative nervous system and the enteric nervous system have been comparatively neglected. In humans the archaic vegetative system has not been researched much at all. It has been only recently that brain researchers have pointed to the importance of the vegetative and unconscious elements of our nervous system for our consciousness (Damasio, 2000). And even more recent is the interest in the connection between the enteric nervous system and the central nervous system (Mayer, 2011; Del Colle et al., 2019; Osadchiy et al., 2019; Iseger et al., 2020).

Similarly, we would venture to surmise that an inclusion of the archaic vegetative system, the even older communication system of the interstitial matrix, in the full picture will also change the way we see ourselves and might introduce a new arsenal of therapeutic options (Schleip et al., 2012; Tozzi, 2015a,b; Langevin et al., 2016; Bordoni and Marelli, 2017).

### The Integration of the Nervous Systems

It is a heuristic principle of systems theory, and an empirical finding of evolutionary biology, that higher order organisms and processes integrate lower order ones into feedback circles and their various branches (Jantsch, 1980; Capra and Luisi, 2014; Ingber et al., 2014; Hands, 2015; Dupré and Nicholson, 2018). While for a long time the doctrine was held that the autonomic and the central nervous system operate in complete independence and have nothing to do with each other, we know today that this is quite wrong. There is a well described central-autonomic network (Beissner et al., 2013). Conscious or unconscious experience can trigger autonomic arousal. This we call stress. Conscious anticipation of action prepares the autonomic system for arousal even before the actual demand on oxygen and glucose begins (Al-Khazraji and Shoemaker, 2018). And conscious activity such as meditation or relaxation can influence autonomic arousal (Tang et al., 2009). This has been called the relaxation response (Benson, 1975). Autonomic processes can influence conscious behavior, such as when we become sleepy, or withdrawn and depressed because of an infection (Maes, 1999). Another example is the sickness behavior associated with immune reactions, when we start feeling tired, withdraw and reduce activity, or the lasting effect autonomically activated immune responses have on behavior and feeling (Mondelli and Vernon, 2019). Conscious behavior can also influence seemingly autonomic processes, such as when listening to music or using imagination to influence recovery rates from surgery (Krucoff et al., 2005), or when the conscious experience of green nature from a hospital window impacts how long patients stay in hospital (Ulrich, 1984). Conscious practice such as relaxation, meditation or psychotherapy might activate the parasympathetic branch of the autonomic nervous system and, via the anti-inflammatory response, influence inflammation, an otherwise quite autonomous process initiated by the immune system (Tracey, 2007; Rosas-Ballina and Tracey, 2009). This might be part of the ubiquitous placebo-effect of all sorts of medical interventions (Pacheco-López et al., 2006). Thus, there are multiple reflex and feedback loops between the CNS and the ANS, both bottom up and top down.

In the same sense, we can expect that the archaic vegetative system (AVS) is integrated into the activities of the other two. But just as the autonomic nervous system has been under-researched compared with the CNS, the AVS has been under-researched compared with both the ANS and the CNS. But we can expect that, similar to the interactions discovered between the ANS and the CNS, there will be connections and interactions between the AVS and both the ANS and the CNS into one integrated communication network (Becker and Selden, 1985). We will point out a potential avenue below.

## Discussion

### The Connective Tissue and the Interstitial Matrix

As pointed out above, the connective tissue or the interstitial matrix forms a coordinated and coordinating system that can be seen as part of the archaic vegetative system (AVS) that was used by simple cellular organisms as the only means of communication. It has been comparatively neglected due to the emphasis on the cell, following Virchow’s cellular pathology. But it comes back full circle when we ask ourselves how the regulating processes and the top down regulation of cell formations operate (Discher et al., 2017; Klement, 2017). For it is through the connective tissue that all cells belonging to, for example, an organ, or a muscle are separated from the rest of the organism and unified as distinct organs at the same time (Langevin et al., 2004, 2007, 2013; Schleip et al., 2012; Myers, 2014; Tozzi, 2015b). And it is through the connective tissue that all cells are connected with the milieu of all other cells and hence can, potentially, communicate. Healthy connective tissue contains sufficient water and electrolytes, and collagen proteins form ordered fiber bundles. It is elastic and conductive chemically as well as electrically. If internal or external influences change this basically sound constitution of the connective tissue, pathological or degenerative processes might be initiated.

Thus, the connective tissue is not only an organ of separation, it is also an organ of communication and connectivity (Schleip and Jäger, 2012; Langevin et al., 2016; Bordoni and Marelli, 2017; Bordoni et al., 2018; Bordoni, 2019). Where the connective tissue forms into visually distinct structures, at the end of muscles or separating whole groups of organs, we speak of fascia that then grow into and combine to tendons, ligaments, etc. And a point can be made that bone tissue also belongs to the fascia (Bordoni, 2019). Where the connective tissue differentiates into ever smaller structures combining and dividing cells, we speak of the interstitial matrix.

**FIGURE 1:**
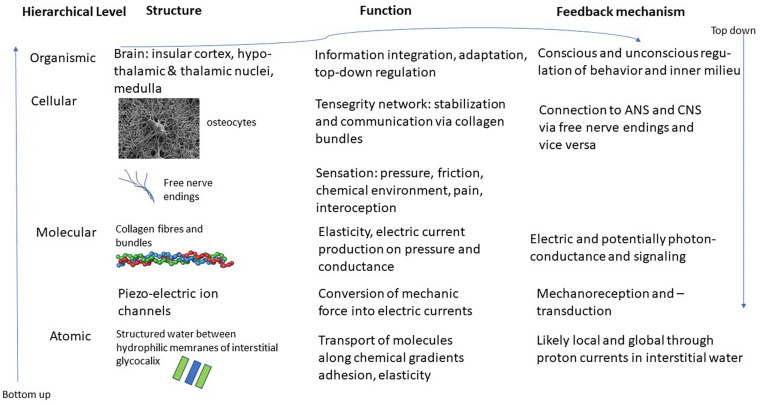
Schematic representation of the various layers and structures of the interstitial matrix, connective tissue and fascia and potential interfaces with the ANS and CNS. Images of osteocyte and collagen bundle from en.wikipedia.org.

The interstitial matrix is formed by macromolecules of proteoglycans, forming what is known as the glycocalyx (Heine and Anastasiades, 1992). These molecules are attached to cell membranes and reach out into the interstitium. Due to their chemical properties – they are hydrophilic – they produce areas known as exclusion zones where water in a quasi-crystalline or ordered form is found (Pollack, 2013). This order is produced through constant intake of radiation, mostly in the form of infrared radiation, whereby the order that is characteristic of life far away from thermodynamic equilibrium, can be maintained (Nicolis and Prigogine, 1977). The special character of this quasi-crystalline or fourth-phase water allows for various effects. For instance, it explains osmotic pressure, as well as strong guided currents of protons in the interstitium. This quasi-crystalline ordered water can also explain some of the mechanical effects, such as the adhesion of organs or muscles to bone or neighboring tissue. This at the same time allows for elasticity and movement that is characteristic of fascia and connective tissue. Whenever chemicals are introduced to this interstitial water it changes its structure and properties locally and potentially also its conductivity. This can lead to transport phenomena from and to micro-vessels or through lymphatic ducts (Langevin et al., 2013). See Figure 1 for a schematic representation of this hierarchical structure.

But in addition to these electro-chemically based potential communication processes, we can also expect multiple interactions with the autonomic and central nervous system (Noguera et al., 2012). The connective tissue is full of nerve receptors. It has 6 times more nerve receptors than muscles, and the spindle receptors of the muscles are also mostly placed at the mechanical interface between muscles and fascia. Thus, the connective tissue is in fact a sense organ (Schleip, 2012; Bordoni, 2019), and it also has contractile properties through myofibrolasts (Schleip et al., 2019). Eighty per cent of all nerve endings in muscles are unmyelinated C-fibers ending in the connective tissue that convey what is called interoception via the spino-thalamic duct to the thalamus and further on to the insular cortex where information is condensed to an image of the inner feeling (Bordoni and Marelli, 2017) and thus available for both conscious and autonomic action (Beissner et al., 2013; Al-Khazraji and Shoemaker, 2018; Naser and Kuner, 2018; Pace-Schott et al., 2019). Thus it becomes, as it were, the basis for Damasio’s somatic consciousness (Damasio, 2000; Schleip and Jäger, 2012).

Collagen is the structural protein of the connective tissue. It is the most abundant protein in the body and stabilizes the connective tissue in fibrillary bundles of a nano-scale structure that is directed. Not only is it directional it also produces electric charges in response to mechanical pressure (Harnagea et al., 2010). This current is always directional within a fibrillary bundle but can run in the opposite direction in a neighboring bundle. This means that there is a very fine tuned mechanic-electric communication system within the connective tissue that is mediated via collagen and its fibrillary structures (Huang and Greenspan, 2012; Langevin et al., 2013). Therefore, the connective tissue has been called a signaling network (Langevin, 2006; Oschman, 2012; Schleip, 2012). We know from collagen that it can exhibit electric semiconductor and photoconductor properties *in vitro*. Whether this is also true *in vivo* we do not know currently. But we do know that it has piezoelectric properties (Vos et al., 2003; Langevin, 2006; Liu et al., 2012). Piezoelectric ion channels in the human organism are responsible for mechanotransduction and mechanoreception, as well as for the sensitivity of baroreceptors (Schrenk-Siemens et al., 2015; Nonomura et al., 2017; Zeng et al., 2018; Burke et al., 2019).

Thus, the potential semiconductor and photoconductor properties in the connective tissue might play an important role that needs to be further elucidated. In view of the fact that important physiological processes utilize either quantum processes, for instance the photosynthetic effect in plants (Collini et al., 2010), or can be analyzed holistically similar to quantum processes (Turvey, 2015), and that proteins exhibit properties that are in a meta-stable critical quantum state (Vattay et al., 2015) it would not really be surprising to find that both the properties of single collagen molecules and the behavior of the connective tissue as a whole supports even more coherent processes than just piezoelectric ones. Indeed, it could recently be shown that certain proteins can become semiconductors and carry large electric charges, likely by a quantum process (Lindsay et al., 2017). And hemoglobin is different from chlorophyll, the molecule supporting photosynthesis, only by the fact that it contains a core of iron instead of magnesia.

A further potential, if not proven, mechanism might be long-range coherence that was theoretically modeled by Fröhlich (Fröhlich and Kremer, 1983). Thereby electromagnetic, or indeed photonic, coherent oscillations can be sustained by biological materials, provided the microstructure and dimension is a multiple of the wavelength that is used for transmission (Hameroff, 1988). It has been postulated that this model can explain the efficiency of catalase reactions that neutralize the hydroxyl radical H_2_O_2_ (Milgrom, 2016), and it might support an as yet hypothetic communication system in the body based on light (Popp, 2006; Ives et al., 2014). But *if* such a system were to be found, the connective tissue with its structure would be the prime candidate for its anatomical and physiological substrate.

Meanwhile let it suffice to enumerate the conventional processes that are already known to be supportive of the connective tissue communication function that can explain a lot of pathophysiology and effects of manual therapy (Myers, 2014; Tozzi, 2015a,b):

(a) There is the *chemical* communication process via the ordered structure of water and its disruption and reorganization due to exchange processes at the cellular level.
(b) There is the communication between the mechanical and chemical environment of the connective tissue and the brain through interoception via free *nerve endings* of unmyelinated C-fibers.
(c) There is the *electromagnetic* communication between the connective tissue and other parts of the body via *piezoelectric* effects mediated by collagen and other protein structures.

Speculatively, but supported by indirect evidence there might be;

(d) A coherent excitation mode of communication via electrons or photons within the tensegrity network (Ingber et al., 2014) of the protein structures of the connective tissue.

## Conclusion: The Vegetative Receptor-Vascular Reflex

Thus, the connective tissue with its interstitial matrix supports at least three, potentially four modes of communication that are partially to be differentiated from the already known communication modes of the nervous systems. These communications might be local, from cell to cell or from cell-groups to cell groups via the interstitium. But they can also be global via the fibroblasts that form a coherent network themselves within the interstitium (Kjaer, 2004; Langevin et al., 2004, 2007, 2013; Kim et al., 2011).

They might be centrally integrated via interoceptive nerve endings and their afferents to the brain (Schleip and Jäger, 2012). And they might be regulative from the interstitium and connective tissue back to the cells (Spencer et al., 2010; Schleip et al., 2019), and from the cells forward to regulating centers elsewhere in the body, be they in the ganglia, organs or even in the brain (Hoheisel et al., 2012; Ingber et al., 2014).

**FIGURE 2:**
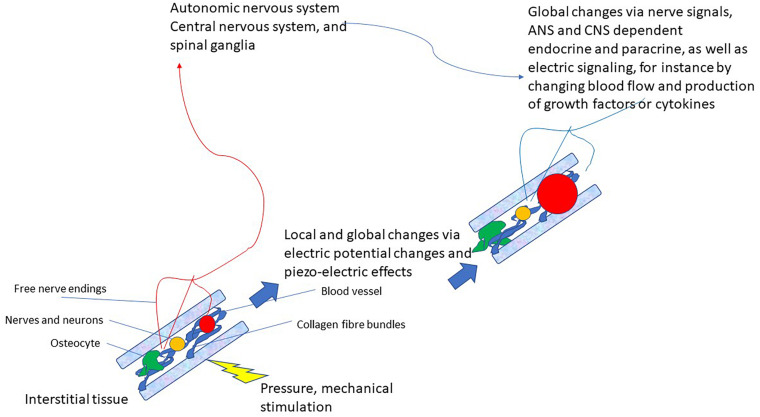
Schematic drawing of the vegetative receptor – vascular reflex (VRVR): mechanical pressure or stimulation is taken up by the mechanosensitive network of collagen fibers, cell-membranes, osteocytes and mechanoreceptors, relayed by local electric currents within the interstitium and by free nerve endings to the ANS/CNS. Thus, global and local changes can be effected that move from local pressure or stimulation to central integration and back to localized changes in the tissue. Also local feedback between nervous tissue (neurons, axons, and ganglia) is possible by this pathway, as they are also embedded in the interstitium.

This latter pathway that very likely also integrates various modes of connective tissue communication yields the basis for a completely new reflex that can be utilized for therapeutic purposes: *the vegetative receptor-vascular reflex (VRVR)*. This is depicted in Figure 2.

Thereby mechanical stimulation of the connective tissue that is frequently employed by various manual therapies, such as connective tissue massage, Rolfing and functional integration, osteopathy and other methods, can lead to changes in set-points thereby reestablishing functionality not only of limbs, muscles and ligaments, but also of organs. It has been already experimentally demonstrated that form follows function: periostal tissue can be converted into chondroitic tissue *in vivo* following a transplantation which also bestows a new function on the periostal tissue (Moukoko et al., 2010). This reflex lends itself to a rational understanding of the well- known effects of manual therapies, and it might turn out to be a reflex with widespread applicability. This warrants further study. We make some suggestions for further research and describe some preliminary empirical findings in the next section.

## A Way Forward: Potential Applications, Empirical Hints, and Experimental Tests

This proposal is of course highly speculative. It is a potential communication route within the body, which, however, needs firm corroboration, ideally through experiments in living organisms. We generated the idea by observing the tissue healing effects produced by a highly gifted manual therapist from Salzburg, Austria, Mohammed Khalifa (MK). Over decades he has developed his own system of manual therapy, which consists of palpating points in the fascia and connective tissue of patients with torn ligaments, but also other patients, usually distally from the locus of pain or trauma. Using slow, pressing and vibrating moves MK stimulates fascia, tendons and ligaments incl. its various receptors following a gradient of resistance and pain toward points of strong responsivity, in terms of subjective pain experienced by the patients, or in terms of palpatory sensations experienced by himself. Using this technique, he has been highly successful in treating high profile athletes, such as world-champions in tennis, rock-climbing, soccer and other sports who had injured themselves by luxating joints or tearing ligaments. As a rule, they were able to resume their sport without surgery or a break immediately after one treatment. We studied MK’s technique in a series of clinical and experimental studies and were able to document the full end-to-end healing of torn cruciate ligaments after 3 months without any reconstitutive surgery in seven of 15 patients documented by magnetic resonance imaging in the first study (Ofner et al., 2014). Our second study showed that a strong control condition of another specialized physiotherapist produced clinically comparable results (Ofner et al., 2018). This points to the fact that MK’s method is likely an approach used by various other therapists. Experimental measurements in controlled studies in patients with anterior cruciate ligament ruptures that were treated once by MK showed that the treatment leads to a higher blood oxygenation immediately after the treatment – measured with near infrared spectroscopy (Litscher et al., 2013b) – and to an adjustment of temperature measured by thermography, likely because of an effect on the vasculature (Litscher et al., 2013a). This demonstrates the immediate effect of connective tissue treatment on the vasculature. Other measurements following MK’s or other, similar physiotherapeutic treatments of injured patients have documented the release of pluripotent stem cells that are the progenitors of tissue repair mechanisms, as well as the release of interleukin-6, norepinephrine, metalloproteinase 9, myoglobin, and cortisol (Stelzer et al., 2015).

It might be possible to study MK’s method in more depth by training other therapists – this is currently being done – and then treating more patients and measuring parameters of interest in those patients comparing them in patients with complete tissue repair against those that do not experience full repair. Parameters of interest might be cell growth factors and pro- and anti-inflammatory cytokines, in addition to stem-cell mobilization. In experimental animal models, chemical and electric gradients in mechanically stimulated connective tissue and peptides or transmitters associated with PIEZO-ion channels could be measured (Schrenk-Siemens et al., 2015; Nonomura et al., 2017; Zeng et al., 2018). Using experimental models it was recently shown that fascia contain myofibroblasts that can contract (Schleip et al., 2019). It should be possible to use similar methods to study causal and mechanistic pathways from mechanical pressure or tension applied to fascia to influence the activity of smooth muscles in the vasculature, either locally through local effects, or globally through central effects. The latter would surely be only possible in live animals. Our study of a vascular response to MK’s therapy (Litscher et al., 2013a,b) gives sufficient rationale for such an investigation. Should our proposal bear out and find experimental and more clinical confirmation, the Vegetative-Receptor-Vascular-Reflex might become a non-invasive therapeutic route for injuries and tissue repair by treating the connective tissue and the fascia manually.

## Data Availability Statement

The original contributions presented in the study are included in the article/supplementary material, further inquiries can be directed to the corresponding author.

## Author Contributions

MO had the conceptual idea for this manuscript, did some of the background research, and wrote parts of the manuscript. HW continued the background research, wrote the first draft of the manuscript, and was responsible for final editing. Both authors contributed to the article and approved the submitted version.

## Conflict of Interest

The authors declare that the research was conducted in the absence of any commercial or financial relationships that could be construed as a potential conflict of interest.
